# Risk of epilepsy in gonadal teratoma: a nationwide population-based study

**DOI:** 10.1038/s41598-023-38255-x

**Published:** 2023-07-11

**Authors:** Seonghoon Kim, Hasung Kim, Jungkuk Lee, Si Baek Lee, Yun Jeong Hong, Yoon-Sang Oh, Jeong Wook Park

**Affiliations:** 1 Department of Neurology, Uijeongbu St. Mary’s Hospital, College of Medicine, The Catholic University of Korea, Seoul, Republic of Korea; 2 Data Science Team, Hanmi Pharm. Co., Ltd., Seoul, Republic of Korea

**Keywords:** Neurology, Epilepsy, Germ cell tumours

## Abstract

Epilepsy is a common neurological disease. Systemic tumors are associated with an increased risk of epileptic events. Paraneoplastic encephalitis related to gonadal teratoma is frequently accompanied by seizures and life-threatening status epilepticus (SE). However, the risk of epilepsy in gonadal teratoma has not been studied. This study aims to investigate the relationship between epileptic events and gonadal teratoma. This retrospective cohort study used the Korean National Health Insurance (KNHI) database. The study population was divided into two study arms (ovarian teratoma vs. control and testicular teratoma vs. control) with 1:2 age and gender-matched control groups without a history of gonadal teratoma or other malignancy. Participants with other malignancies, neurologic disorders, and metastatic brain lesions were excluded. We observed the occurrence of epileptic events during the observation period (2013–2018) and investigated the risk of epileptic events in each gonadal teratoma group compared to controls. In addition, the influence of malignancy and tumor removal was investigated. The final analysis included 94,203 women with ovarian teratoma, 2314 men with testicular teratoma, and controls. Ovarian teratoma is associated with a higher risk of epilepsy without SE (HR, 1.244; 95% CI 1.112–1.391) and epilepsy with SE (HR, 2.012; 95% CI 1.220–3.318) compared to the control group. The risk of epilepsy without SE was higher in malignant ovarian teratoma (HR, 1.661; 95% CI 1.358–2.033) than in benign (HR, 1.172; 95% CI 1.037–1.324). Testicular teratoma did not show significant relations with epileptic events. The risk of epileptic events showed a tendency to decrease after removing the ovarian teratoma. This study found that ovarian teratoma is associated with a higher risk of epileptic events, especially in malignant tumors, whereas testicular teratoma did not show significant differences in epileptic events compared to the control group. This study adds to the current understanding of the association between gonadal teratoma and epileptic events.

## Introduction

Epilepsy is a common neurological disease with a cumulative lifetime incidence of 7.60 per 1000 individuals in the general population^1^. Systemic tumors can occur concurrently with epilepsy, and 13% of systemic tumor patients experience seizures^2^. The most common cause of epilepsy in tumors is intracranial metastasis (50%), but paraneoplastic syndrome-related immunologic mechanisms have also been frequently reported^2,3^.

Gonadal teratoma is the most common germ cell tumor that originates from pluripotent primitive germ cells^4,5^. They are mostly asymptomatic or present non-specific symptoms such as abdominal pain and distension^5,6^. Epilepsy in gonadal teratoma is frequently associated with paraneoplastic encephalitis, with seizures occurring as an initial symptom^7^. Paraneoplastic encephalitis in gonadal teratomas is related to immunologic mechanisms originating from tumor cells, and anti-N-methyl D-aspartate receptor encephalitis (anti-NMDARE) and anti-MA2 encephalitis are well-known examples^7^. In anti-NMDARE, seizures occur frequently in 70% of cases. Among patients with seizures, 35% experience status epilepticus (SE) and 21% experience refractory status epilepticus^8^. Anti-Ma2 encephalitis presents limbic encephalitis, brain stem symptoms, and seizures^7^. The anti-NMDAR antibody and anti-MA2 antibody have been identified and used for diagnosis and management^7^.

Severe paraneoplastic encephalitis can result in death or severe neurologic sequelae^9,10^. However, about 80% of patients may improve with proper immunotherapy and removal of gonadal teratoma. Earlier treatment and the absence of intensive care unit management are predictive factors of a good prognosis^8^. Therefore, early diagnosis and appropriate treatment are crucial for the prognosis of autoimmune encephalitis. This suggests that patients with gonadal teratoma may be more closely associated with seizures than the general population. However, the prevalence of epilepsy in gonadal teratoma has not been reported. Our study investigated the risk of epilepsy in ovarian and testicular teratoma. Additionally, we investigated the effect of malignancy and tumor removal on the risk of epilepsy in gonadal teratoma. We used a population-based cohort that included medical information from the Korean National Health Insurance (KNHI) database.

## Results

### Demographics and characteristics

**Figure 1:**
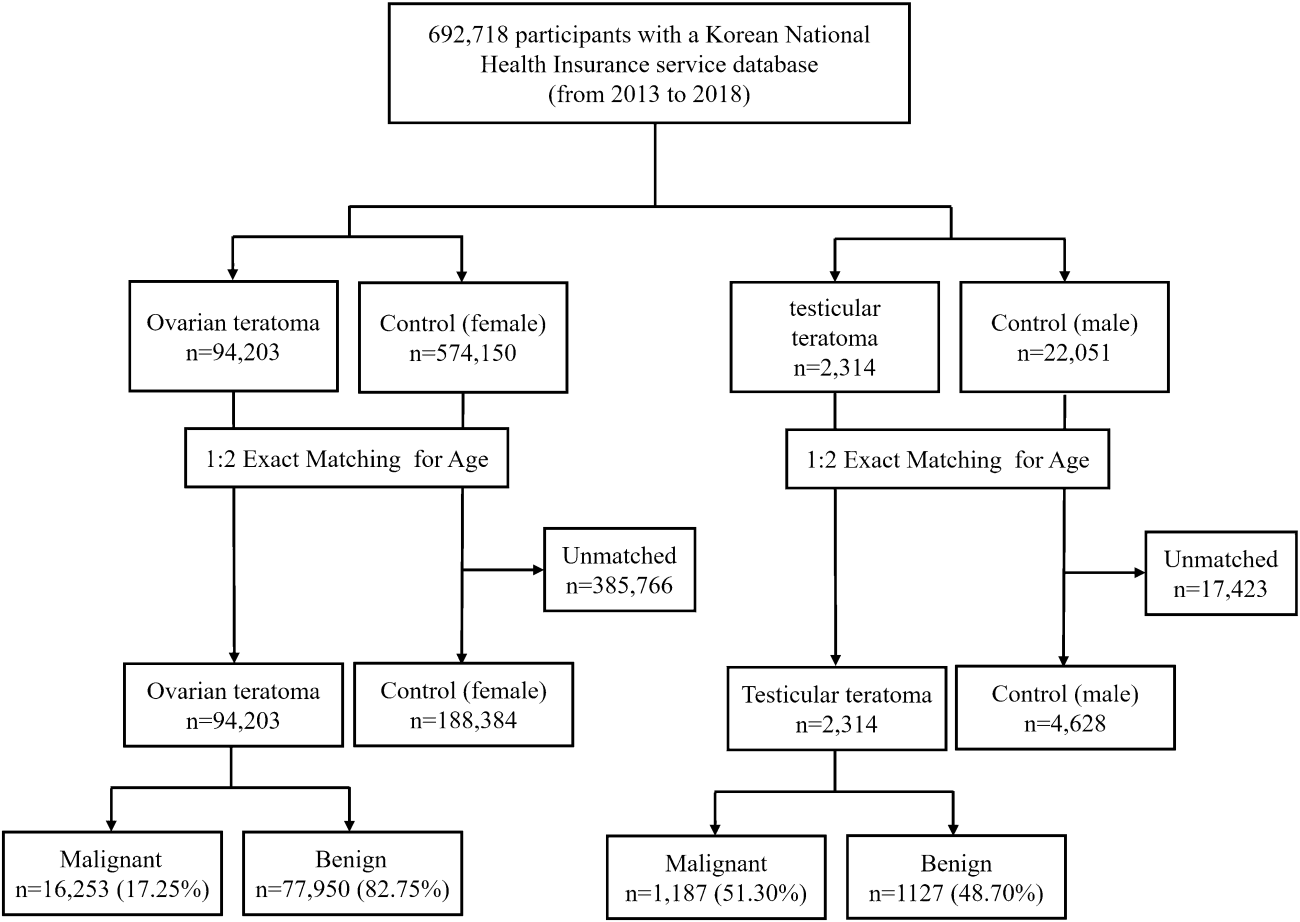
Flow chart of the study process.

A total of 692,718 participants were enrolled in this study using KNHI data between 2013 and 2018. After 1:2 exact matching for age, 94,203 women (malignant ovarian teratoma, 16,253, 17.25%) with ovarian teratoma and 188,304 controls, as well as 2314 men (malignant testicular teratoma, 1187, 51.30%) with testicular teratoma and 4628 controls were included in the analysis (Fig. 1). The ovarian teratoma group had a higher frequency of dyslipidemia, ischemic heart disease, and a lower frequency of hypertension compared to the control group. In the testicular teratoma group, dyslipidemia and ischemic heart disease were more prevalent than in the control group. Tumor resection was performed in 23,770 ovarian teratoma patients and 539 testicular patients (Table 1).

**Table 1:** Demographics and population characteristics of teratoma and age-matched control groups.

	Control	Ovarian teratoma	p	Control	Testicular teratoma	p
Total (n)	188,384	94,203		4628	2314	
Age (years)	40.99 ± 11.83	40.99 ± 11.83		41.68 ± 14.97	41.68 ± 14.97	
Hypertension (HBP)	31,181 (16.55)	15,176 (16.11)	0.0028	1176 ± 25.41 (25.41)	594 (25.67)	0.8152
Diabetes (DM)	34,659 (18.45)	17,420 (18.49)	0.7920	1009 ± 21.8 (21.8)	522 (22.56)	0.4737
Dyslipidemia	76,357 (40.53)	38,744 (41.13)	0.0024	1896 ± 40.97 (40.97)	1032 (44.6)	0.0039
Ischemic heart disease	15,079 (8.0)	7912 (8.40)	0.0003	557 ± 12.04 (12.04)	320 (13.83)	0.0340
Malignancy		16,253 (17.25)			1187 (51.30)	
Tumor resection		23,770 (25.23)			539 (23.29)	

Data are presented as n (%) or mean ± standard deviation (SD).

### Prevalence of epileptic events in gonadal teratoma

#### Epileptic events in ovarian teratoma

Overall, the ovarian teratoma group did not show a significant difference in total epileptic events, epilepsy with SE, and epilepsy without SE compared to the control group (p = 0.1148). To investigate the differences in epileptic events among the control, benign, and malignant ovarian teratoma groups, we conducted further analyses. The prevalence of total epileptic events (p = 0.0002) and epilepsy without SE (p = 0.0004) was significantly different among the groups. Subgroup analysis revealed that the malignant ovarian teratoma group had a significantly higher prevalence of total epileptic events and epilepsy without SE. The benign ovarian teratoma group did not show a significant difference (Table 2).

**Table 2:** The prevalence of total epileptic events, epilepsy with SE, and epilepsy without SE between total gonadal teratoma and control groups.

	Total	Total epileptic event		Epilepsy without SE		Epilepsy with SE	
	n	n (%)	p	n (%)	p	n (%)	p
Total ovarian teratoma	94,203	502 (0.53)	0.1148	474 (0.5)	0.2191	28 (0.03)	0.0771
Control	188,384	920 (0.49)^a^	0.0002	884 (0.47)^a^	0.0004	36 (0.02)	0.1407
Benign ovarian teratoma	77,950	389 (0.50)^a^		367 (0.47)^a^		22 (0.03)	
Malignant ovarian teratoma	16,253	113 (0.70)		107 (0.66)		6 (0.04)	
Total testicular teratoma	2314	19 (0.82)	0.4174	16 (0.69)	0.8343	3 (0.13)	0.0143
Control	4628	30 (0.65)	0.4940	30 (0.65)	0.5158	0 (0.00)	0.037
Benign testicular teratoma	1127	11 (0.98)		10 (0.89)		1 (0.09)	
Malignant testicular teratoma	1187	8 (0.67)		6 (0.51)		2 (0.17)	

The prevalence of all epileptic events compared between control, malignant, and benign gonadal teratoma groups. Data are presented as n (%). Post-hoc analysis was performed between control, benign, and malignant teratoma groups ^a^ indicates a non-significant difference between groups based on the Bonferroni comparison test.

The total ovarian teratoma group has a statistically significant higher risk of epilepsy without SE (HR, 1.244; 95% CI 1.112–1.391) and epilepsy with SE (HR, 2.012; 95% CI 1.220–3.318) compared to the control group. The risk of epilepsy without SE was higher in malignant ovarian teratoma (HR, 1.661; 95% CI 1.358–2.033) than in benign (HR, 1.172; 95% CI 1.037–1.324). Both the benign and malignant ovarian teratoma groups had a higher risk of epilepsy without SE than the control group. The risk of epilepsy with SE was higher in both the benign (HR, 1.955; 95% CI 1.141–3.347) and malignant ovarian teratoma (HR, 2.520; 95% CI 1.051–6.042) groups compared to the control group (Table 3).

**Table 3:** Hazard ratios of epilepsy without SE and epilepsy with SE among patients with teratoma compared with age-matched controls.

	Total	Number of events	Number of Person-years (10,000)	Incidence Rate	Hazard ratio (95% CI)	
					Model 1	Model 2
Epilepsy without SE						
Control (female)	188,384	884	613,163.00	14.42	1 (Ref.)	1 (Ref.)
Total ovarian teratoma	94,203	474	255,996.10	18.52	1.180 (1.055,1.320)*	1.244 (1.112,1.391)*
Benign ovarian teratoma	77,950	367	218,835.5	16.77	1.070 (0.948,1.209)	1.172 (1.037,1.324)*
Malignant ovarian teratoma	16,253	107	2892.93	28.79	1.871 (1.530,2.288)*	1.661 (1.358,2.033)*
Control (male)	4,628	30	14,135.08	21.22	1 (Ref.)	1 (Ref.)
Total testicular teratoma	2,314	16	5820.11	27.49	1.142 (0.622,2.096)	1.142 (0.622,2.097)
Benign testicular teratoma	1,127	10	2927.18	34.16	1.427 (0.698,2.920)	1.414 (0.691,2.895)
Malignant testicular teratoma	1,187	6	2892.93	20.74	0.857 (0.357,2.059)	0.873 (0.363,2.100)
Epilepsy with SE						
Control (female)	188,384	36	613,867.00	0.59	1 (Ref.)	1 (Ref.)
Total ovarian teratoma	94,203	28	257,044.00	1.09	1.892 (1.149.,3.116)*	2.012 (1.220,3.318)*
Benign ovarian teratoma	77,950	22	219,687	1.00	1.743 (1.020,2.967)*	1.955 (1.141,3.347)*
Malignant ovarian teratoma	16,253	6	37,353.28	1.61	2.805 (1.175,6.700)*	2.520 (1.051,6.042)*
Control (male)	4,628	0	14,164.76	0.00	1 (Ref.)	1 (Ref.)
Total testicular teratoma	2,314	3	5851.56	5.13	–	–
Benign testicular teratoma	1,127	1	2947.332	3.39	–	–
Malignant testicular teratoma	1,187	2	2904.23	6.89	–	–

The incidence rates are calculated per 10,000 person-years. Model 1 is an unadjusted result. Model 2 adjusted for hypertension, diabetes, dyslipidemia, and ischemic heart disease.

#### Tumor resection and epileptic events in ovarian teratoma

In benign ovarian teratoma, the risk of epilepsy with SE did not differ from control at 1 year after tumor resection. The risk of epilepsy without SE decreased after tumor resection and did not differ from the control at 6 years.

**Figure 2:**
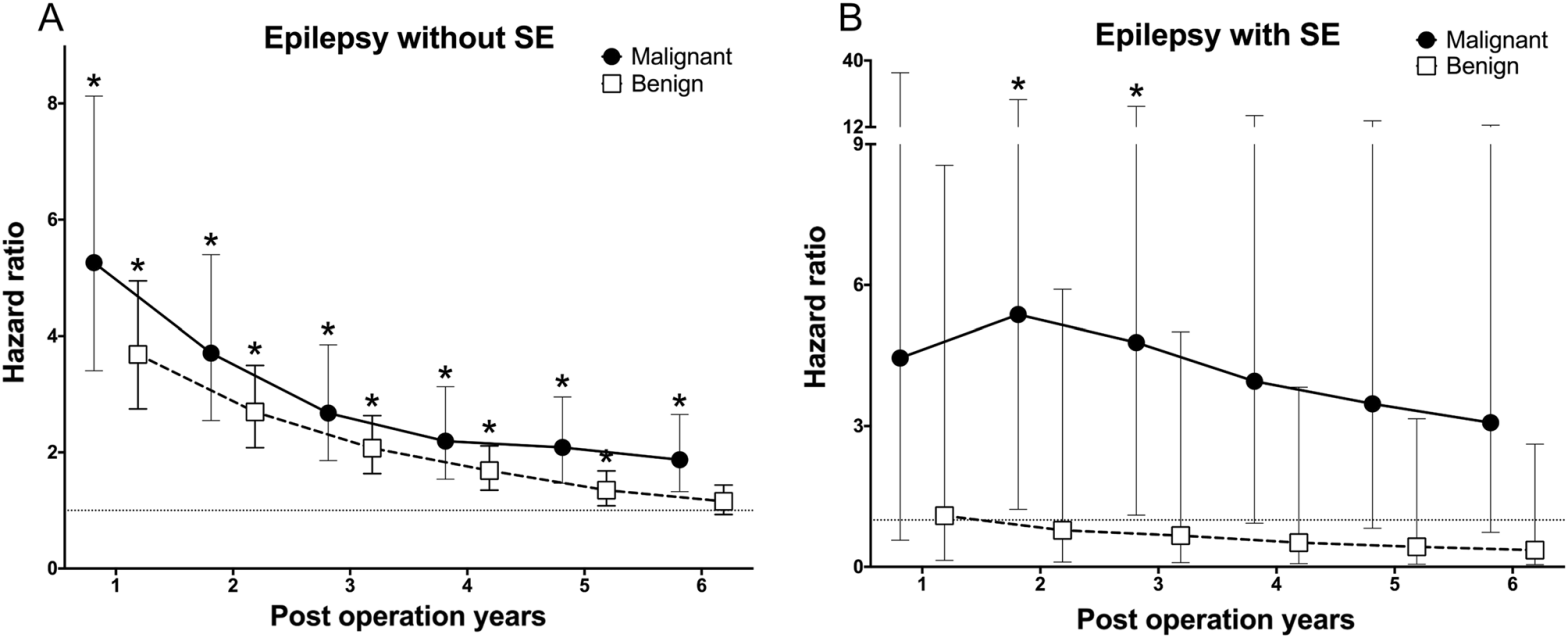
The hazard ratio for epilepsy without SE (**A**) and epilepsy with SE (**B**) according to the post-operation year for each benign and malignant ovarian teratoma group compared with an age-matched control group. Hazard ratios adjusted for hypertension, diabetes, dyslipidemia, and ischemic heart disease. *Indicates statically significant result compared to normal control.

In malignant ovarian teratoma, the risk of epilepsy with SE did not differ from the control at 4 years after tumor resection. The risk of epilepsy without SE was higher than in the control group during the observation period but showed a decreasing trend over time (Fig. 2).

#### Epileptic events in testicular teratoma

The total testicular teratoma group did not show a significant difference in total epileptic events (p = 0.4174) and epilepsy without SE compared to the control group (p = 0.8348). However, the prevalence of epilepsy with SE was higher in the total testicular teratoma group than in the control group (p = 0.0143). To investigate the differences in epileptic events among the control, benign, and malignant testicular teratoma groups, we conducted further analyses. Although the prevalence of epilepsy with SE significantly differed between control, benign, and malignant teratoma (p = 0.037), subgroup analysis did not show significance (Table 2). In all testicular teratoma groups, the risks of epilepsy with SE and epilepsy without SE were not significantly different from the control group (Table 3).

## Discussion

This is a nationwide population-based retrospective cohort study that has investigated the risk of epileptic events in gonadal teratoma. Our main findings show that ovarian teratoma is associated with a higher risk of epileptic events. The risk of an epileptic event is significantly higher in malignant than in benign ovarian teratoma. Testicular teratoma did not show a significant correlation with the risk of epileptic events. Our analysis also revealed that tumor removal significantly reduces the risk of epileptic events in the ovarian teratoma group, with a gradual decrease in risk observed during the postoperative observation period.

### Epileptic events in ovarian teratoma

In patients with tumors, epilepsy can be caused by intracranial metastasis, medication, metabolic issues, or immunologic mechanisms^2^. In our study design, we excluded cases with brain metastasis and other brain lesions. Moreover, the vast majority of gonadal teratomas are benign, and only 1–2% of malignant tumors show intracranial metastasis, suggesting that the possibility of epilepsy due to intracranial metastasis in our study is low^6,11^. Some patients with malignant ovarian teratoma may receive chemotherapy, which can cause seizures through neurotoxicity^12^. Our results showed a higher risk of epileptic events in benign and malignant ovarian teratoma compared to the control group. Therefore, metastasis, medication, and metabolic issues alone may not fully explain the occurrence of epileptic events.

Ovarian teratoma has been associated with paraneoplastic encephalitis, which can cause seizures, and this suggests involvement of immunologic mechanisms^8^. One well-known cause of paraneoplastic encephalitis that can lead to seizures in ovarian teratoma is anti-NMDARE^8^. In ovarian teratoma, anti-NMDAR antibodies derived from neuroglial tissue of gonadal tumors can induce neuronal excitatory neurotransmission imbalances^13^. Purified anti-NMDAR antibodies from patients with paraneoplastic encephalitis have been shown to induce seizures in mice^14^. Interestingly, various ovarian tumors without encephalitis also show B-cell infiltration and autoantibodies. The infiltrated B-cells of ovarian tumors can have pro- and anti-tumor responses and have various effects on progression and treatment^15^. These findings suggest that the humoral immune reaction may cause seizures in ovarian teratoma. However, our data did not include the titer of paraneoplastic antibodies or pathological findings, so it was not possible to confirm an association by an immune mechanism. Therefore, there is a need for careful interpretation of our findings.

### Epileptic events significantly increase in malignant ovarian teratoma compared with benign

In our study, the risk of epilepsy without SE were significantly higher in the malignant ovarian teratoma group compared with the benign group. Several factors can cause epileptic events in malignant ovarian teratoma. Although brain metastasis in malignant teratoma is very rare and was defined as an exclusion criterion in our study, the possibility of some unconfirmed brain metastases could be considered. Some malignant ovarian teratomas require chemotherapy which may induce seizures^12^. The most common regimens include bleomycin, etoposide, and cisplatin^12^. Cisplatin toxicity can cause seizures and electrolyte imbalances, typically occurring 5–15 days after administration^2^. However, an immunological mechanism can also be considered. The neoplastic transformation of oocytes induces the epitope expression related to paraneoplastic antibody^16^. Therefore, it is believed that malignant ovarian teratoma may induce more immune reactions related to epilepsy. In the general population, mature teratoma (MTs), a benign tumor, accounts for 95% of all ovarian teratomas, and immature teratoma (ITs), a malignant tumor, accounts for 1%^17,18^. In paraneoplastic encephalitis with ovarian teratoma, MT is known to be the most common^18^. However, several studies have reported that the incidence of malignant tumors in ovarian paraneoplastic syndrome is higher than in the general population. In NMDARE with ovarian teratoma, the frequency of ITs was 14–20%^19–21^. Our results suggest that pathological findings may be related to the risk of epilepsy in ovarian teratoma.

### The influence of tumor resection on epilepsy in ovarian teratoma

During the observation period, the risk of epilepsy following ovarian tumor resection decreased over time, and this trend was more pronounced in benign ovarian teratoma. Exactly how the removal of ovarian teratoma reduces the risk of seizures is not completely understood, but the removal of the tumor may reduce the risk factors associated with epilepsy such as intracranial metastasis and chemotherapy. Additionally, tumor resection can reduce an improper immunological reaction. In most paraneoplastic syndromes, primary tumor removal is a crucial treatment option^7,22^. Tumor removal has been shown to increase the recovery rate and reduce the risk of relapse in paraneoplastic encephalitis associated with ovarian teratoma^18,23^. Early intervention and removal of even a small teratoma are recommended for paraneoplastic encephalitis^18^. Further observational studies are required to confirm the association between tumor resection and epileptic risk in ovarian teratoma.

### Epileptic events in testicular teratoma

Contrary to ovarian teratoma, the risk of epileptic events in testicular teratoma was not significantly increased compared to the control group. Several factors may account for these results. First, testicular teratoma has different clinical and pathological characteristics compared to ovarian teratoma. Testicular teratoma is frequently seen in young men between 18 and 35 years of age or in children under the age of five. It is known that childhood teratoma shows a pathogenesis similar to that of ovarian teratoma^24^. However, since we mainly enrolled adult males with testicular teratoma, there may be differences compared to ovarian teratoma. The second possible factor is the immunological mechanisms involved. The seizure is a common symptom of anti-NMDARE. However, the prevalence of anti-NMDARE is nine times higher in women than in men^25^. The anti-MA2 encephalitis, most frequent in testicular teratoma, shows limbic encephalitis and ataxia, but seizures are not frequent^7^. Third, hormonal factors may have influences on the immunologic response. Estrogen mediates humoral immunity which exacerbates the autoimmune disease, whereas testosterone has a protective effect^26–28^.

There were some limitations to our study. This large population-based study analyzed information from the KNHI database and enrolled participants with gonadal teratoma based on ICD-10 codes. However, the KNHI database did not include detailed pathological or imaging results which may have limited our ability to make accurate diagnoses or exclude potential confounding factors. Additionally, although the KNHI is responsible for registering and managing patients with malignant tumors, we were unable to verify the accuracy of those registrations or exclude the influence of chemotherapy or metabolic problems on our results. Furthermore, we were unable to investigate the occurrence of paraneoplastic encephalitis in our participants due to the lack of corresponding ICD-10 codes in the KNHI database. Moreover, as KNHI does not yet cover testing for paraneoplastic antibodies, we were unable to check the titer of paraneoplastic antibodies, which may have provided additional insights into the underlying mechanisms of the observed epileptic events. These limitations highlight the need for future studies that incorporate more detailed clinical and laboratory data to further explore the relationship between gonadal teratoma and epileptic events.

Our results showed that ovarian teratoma is associated with an increased risk of epileptic events, whereas testicular teratoma does not show a significant increase in risk. Furthermore, malignant ovarian teratoma is associated with a higher risk of epilepsy than benign teratoma. Tumor removal significantly reduces the risk of epileptic events and a gradual decrease in the risk was observed during the postoperative period. Our findings contribute to the current understanding of the relationship between gonadal teratoma and epileptic events. However, the underlying mechanisms of these findings remain unknown and require further investigation.

## Methods

### Data source and ethical approval

We obtained data from the KNHI database. The KNHI program provides medical services for the entire Korean population, resulting in a sample size of over 50 million individuals^29^. The KNHI database includes all claims data and lists diagnoses by International Classification of Disease, Tenth Revision (ICD-10) codes.

This study was approved by the Institutional Review Board of the Korean National Institute for Bioethics Policy (NHIS-2021-1-226) and conducted in accordance with the principles of the Declaration of Helsinki. The study used anonymized and de-identified data, and therefore informed consent was not required. Additionally, the Institutional Review Board of the Catholic University of Korea (no. UC20EISE0003) approved the study and waived the requirement for informed consent.

### Study design and study population

This study is a nationwide population-based retrospective cohort study using the KNHI database. Gonadal teratomas were defined using the ICD-10 diagnostic codes for ovarian (D279, C569) and testicular teratoma (D297, C629) in the KNHI database. Participants with systemic malignancies other than gonadal teratoma, neurologic disorders, and metastatic brain lesions were excluded. The study was conducted in two study arms (ovarian teratoma vs. control and testicular teratoma vs. control), with control groups defined as 1:2 gender and age-matched participants without a previous history of gonadal teratoma or other malignancy. Malignant and benign groups were divided according to the ICD-10 codes (C569, C629) in each teratoma group. Newly developed epileptic events were investigated in both study arms during the observation period (between 2013 and 2018). The risk of epileptic events was also evaluated between malignancy and benign teratoma groups.

Epileptic events were defined as epilepsy without SE and epilepsy with SE, according to the diagnostic ICD-10 code (Epilepsy without SE: G400-402, G406-409; Epilepsy with SE: G410-G419). In the KNHI database, epileptic events may include a single unspecified seizure attack or convulsive syncope. To increase diagnostic accuracy, epileptic events were defined as a participant who received a diagnostic code and anti-seizure medication (ASM; valproic acid, phenytoin, phenobarbital, carbamazepine, oxcarbazepine, ethosuximide, lamotrigine, topiramate, zonisamide, levetiracetam, gabapentin, pregabalin, vigabatrin, perampanel, clonazepam, clobazam, rufinamide, and lacosamide) for more than three months. The exclusion criteria for epileptic events are as follows. Participants without a diagnostic code, regardless of whether they were taking an ASM. Participants with a diagnostic code but not taking an ASM. Participants with a diagnostic code taking an ASM for less than three months. In Korea, SE is classified as a "Rare and Incurable Disorder," which entitles patients with SE to receive additional medical insurance benefits. The diagnosis code for SE is assigned only when the diagnostic criteria specified by the Korea National Health Insurance (KNHI) are met, ensuring the reliability of the diagnosis. The KNHI diagnostic criteria for SE require seizures to last for more than five minutes or repeated seizures without recovery of consciousness. Although the subtype of SE cannot be identified, it is classified as a severe symptom of epilepsy. The risk of epileptic events between the teratoma with tumor resection and the control groups was compared to investigate the influence of tumor resection. Tumor resection in teratoma patients was confirmed using the KNHI reimbursement code (oophorectomy: R4421, R442101, R4423-R4428; orchiectomy: R3851-R3853, R3861, R3862).

### Statistical analysis

This study used the Student's t-test for continuous variables and the chi-square test for binary and categorical variables to compare the group characteristics and epileptic events among study groups. The incidence rate of all epileptic events compared between control, malignant, and benign gonadal teratoma groups. Post-hoc analysis was performed based on the Bonferroni comparison test. The incidence rate and hazard ratio of epileptic events were calculated for the teratoma and control groups, with an incidence rate of 10,000 person-years in each group. The hazard ratio (HR) was analyzed using Cox’s proportional hazard regression models with a 95% confidence interval (CI) to determine the relative risk of each factor compared to the reference. Model 2 was used to control for confounding factors, including hypertension, diabetes, dyslipidemia, and ischemic heart disease. The same statistical method was used to compare the malignant and benign teratoma groups. The risk of epilepsy in malignant and benign teratoma patients who underwent tumor resection was compared with control over time using the Cox proportional hazard regression model. All statistical analyses were performed using SAS software (ver. 9.4; SAS Institute, Cary, NC, USA).

### Ethical approval

This study was approved by the Institutional Review Board of the Korean National Institute for Bioethics Policy (NHIS-2021-1-226) and conducted in accordance with the principles of the Declaration of Helsinki. The study used anonymized and de-identified data, and therefore informed consent was not required. Additionally, the Institutional Review Board of the Catholic University of Korea (no. UC20EISE0003) approved the study and waived the requirement for informed consent. We confirm that we have read the Journal’s position on issues involved in ethical publication and affirm that this report is consistent with those guidelines.

## Data Availability

Data cannot be shared publicly due to identity protection. Raw data are available from the National Health Insurance Date Sharing Service (https://nhiss.nhis.or.kr/bd/ab/bdaba000eng.do) for researchers who meet the requirements for access to confidential data.
